# Autofocusing MALDI MS imaging of processed food exemplified by the contaminant acrylamide in German gingerbread

**DOI:** 10.1038/s41598-023-32004-w

**Published:** 2023-04-03

**Authors:** Oliver Wittek, Andreas Römpp

**Affiliations:** Bioanalytical Sciences and Food Analysis, University of Bayreuth, Universitaetsstrasse 30, 95447 Bayreuth, Germany

**Keywords:** Imaging studies, Mass spectrometry

## Abstract

Acrylamide is a toxic reaction product occurring in dry-heated food such as bakery products. To meet the requirements laid down in recent international legal norms calling for reduction strategies in food prone to acrylamide formation, efficient chromatography-based quantification methods are available. However, for an efficient mitigation of acrylamide levels, not only the quantity, but also the contaminant’s distributions are of interest especially in inhomogeneous food consisting of multiple ingredients. A promising tool to investigate the spatial distribution of analytes in food matrices is mass spectrometry imaging (MS imaging). In this study, an autofocusing MALDI MS imaging method was developed for German gingerbread as an example for highly processed and instable food with uneven surfaces. Next to endogenous food constituents, the process contaminant acrylamide was identified and visualized keeping a constant laser focus throughout the measurement. Statistical analyses based on relative acrylamide intensities suggest a higher contamination of nut fragments compared to the dough. In a proof-of-concept experiment, a newly developed *in-situ* chemical derivatization protocol is described using thiosalicylic acid for highly selective detection of acrylamide. This study presents autofocusing MS imaging as a suitable complementary method for the investigation of analytes’ distributions in complex and highly processed food.

## Introduction

Acrylamide is a toxic reaction product, which is under debate due to its presence in a variety of heated foodstuffs, especially in carbohydrate-rich food^1^. As a Maillard-reaction product it is formed mainly during dry heating above 120 °C (frying, baking or roasting) of food containing its precursors, namely the amino acid asparagine and reducing sugars^2,3^. The cause of increasing concern about the presence of acrylamide in food lies in its toxic and carcinogenic properties, mainly exerted by its epoxidation product glycidamide^4,5^. Acrylamide is declared as “probably carcinogenic to humans” by the International Agency for Research on Cancer (IARC)^6^ and the harmonized classification and labelling (CLP) approved by the European Union lists the substance as carcinogenic, mutagenic and toxic to reproduction (Reg. (EC) 1232/2008)^7^. In the context of food and with respect to human studies^8^, acrylamide’s carcinogenicity is, however, considered inconclusive by the European Food Safety Authority’s Panel on Contaminants in the Food Chain (EFSA CONTAM Panel)^9^. In the light of reports on an endogenous level of acrylamide in the human bloodstream^10^ and recent scientific recommendations towards the definition of a tolerable daily intake (TDI)^11^, the discussion about acrylamide in food and the necessity of mitigation measures has been sparked once more. Nevertheless, since acrylamide is considered a food contaminant, international legislation introduced guidelines and strategies to reduce its amount in foodstuffs^12^. While the US Food and Drug Administration (FDA) released a Guidance document for the industry in 2016^13^, the EU passed Regulation (EU) 2017/2158^14^, which entered into force on April 11, 2018. Among the food listed in these legal norms, gingerbread is a prominent example since it is prone to acrylamide formation during baking^15,16^. Next to possible approaches to reduce acrylamide in gingerbread, a benchmark level of 800 µg/kg was set by the Commission^17^. Traditional German gingerbread (‘Elisenlebkuchen’) is an exquisite spiced sweet product consisting of less than 10% cereal products and at least 25% almonds, walnuts and/or hazelnuts^18^, thus providing the necessary acrylamide precursors. Another, less prominent, example for potentially acrylamide-rich food is pancakes. Although not falling within the scope of the respective Regulation, pancakes might contain significant levels of acrylamide and could therefore be relevant contributors to the dietary exposure to acrylamide, which is why they are listed in Commission Recommendation (EU) 2019/1888 as food to be monitored regularly regarding the presence of acrylamide^19^. Since pancakes are often prepared by customers at home, i.e., in uncontrolled conditions compared to industrial baking, the investigation of acrylamide formation is of high interest from a food safety point of view. In addition, pancakes constitute a suitable model system for mass spectrometry imaging investigation of acrylamide formation in food.

To reliably determine the amounts of acrylamide in food, powerful analytical methods are needed. Currently, mass spectrometry-based methods after Carrez precipitation, Quechers extraction and chromatographic separation are most common for acrylamide analysis, some also include derivatization strategies prior to or after chromatographic separation^8,20^. However, these techniques require homogenization and provide no information on the spatial distribution of acrylamide throughout the food sample. For the development of efficient mitigation measures tailored to the specific food product, it is not only the mere quantity of acrylamide, which is of interest to manufacturers, but also knowledge about the distribution and possible hotspots. A promising analytical tool to fill this gap is mass spectrometry imaging (MS imaging). MS imaging is a rapidly evolving technique in (bio-)analytical chemistry which links mass spectrometric data to defined locations throughout the surface of an analyzed biological sample. In medicine as well as in animal and plant biology, MS imaging has steadily developed into a sophisticated technology with numerous applications^21–24^. The rapid expansion of MS imaging in these fields has also sparked applications in food analysis. The majority focused on the localization of constituents in unprocessed fresh food using matrix-assisted laser desorption/ionization (MALDI) as ionization technique^25^. Moreover, fresh food samples have also been investigated using desorption electrospray ionization (DESI)^26^, laser-ablation electrospray ionization (LAESI)^27^ and secondary ion mass spectrometry (SIMS)^28^. Advancements of MS imaging in the field of food analysis were reviewed by Yoshimura et al.^29^. In recent years, also processed food with an altered texture compared to the respective raw product became subject to MS imaging studies, such as dry-cured ham^30^ and roasted coffee beans^31^. Due to alterations of the native cell structure during processing steps, such as cooking, baking, roasting or drying, processed food usually poses a greater challenge regarding sample preparation. Well-established preparation protocols might need to be adapted or even replaced by suitable alternatives, especially when attempting to image low abundant analytes, such as acrylamide. For instance, cryosectioning, as the standard method for the preparation of sample sections for MS imaging, is not suitable for inhomogeneous samples such as German gingerbread. Cryosectioning has been successfully applied to obtain thin sections of food both of animal and plant origin, such as dried ham muscle^32^ or wheat grains^22^. However, its success often depends on a compact tissue structure or at least a firm sample texture. Thus, for samples with an altered and delicate texture, an alternative sectioning method is needed. Apart from the challenge of preparing processed food samples and successfully visualizing low abundant components with MALDI MS imaging, the selectivity of acrylamide detection constitutes another challenge. Commonly applied measures to ensure high selectivity in MS imaging experiments are high mass accuracy and *in-situ* MS/MS experiments^33^. In liquid chromatography methods, another common tool to enhance selectivity is derivatization of the target analytes prior to or following chromatographic separation^34^. For acrylamide detection in HPLC, derivatization protocols using D-cysteine^35,36^, 2-napththalenethiol^37,38^ and thiosalicylic acid (TSA)^39,40^ have been published. All derivatization reagents contain a thiol group which reacts with the double bond of acrylamide to form a Michael adduct. Analogous to HPLC, various protocols for on-tissue chemical derivatization (OTCD) have been described for MS imaging methods of both animal- and plant-based samples^41,42^. However, existing protocols are almost exclusively used to enhance the ionization yield of poorly ionizable analytes^43^. Gaining higher selectivity has scarcely been the primary motivation for the development of derivatization strategies in MS imaging so far.

We have recently published an extensive study on MS imaging experiments tailored to both fresh and processed food including first MS imaging results of the additive natamycin and the contaminant acrylamide in processed food^44^. In the respective study, we describe the proof-of-concept for detection of acrylamide in a gingerbread cross-section with a lateral resolution of 200 µm using a fixed MALDI-laser. In the present study, we go one step further by using an additional autofocusing laser (AF-laser), which allows adjusting the laser focus for each pixel position and thereby keep measurement conditions constant throughout the entire analysis^45^. By resetting the focus prior to each pixel acquisition, enhanced signal intensities can be obtained resulting in MS images with higher contrast. In addition, measurement in AF-mode allows for smaller pixel sizes, in this case 100 µm. The resulting higher number of pixels per area produces a larger data basis and enables more profound (statistical) analyses on analyte distributions, which are presented in our study by endogenous constituents and the contaminant acrylamide in German gingerbread. All measurements are based on a sample preparation workflow for MS imaging of brittle, inhomogeneous food samples, which is described in detail below. Additionally, we consider quality control aspects regarding the possible acrylamide formation during sample preparation and analysis. In this context, we identified the critical steps for artifact formation and conducted complementary measurements. To increase the selectivity of acrylamide detection beyond accurate mass and *in-situ* MALDI-MS/MS, a new derivatization method for acrylamide on the surface of pancakes was developed.

## Results and discussion

### Development of a sample preparation workflow

**Figure 1:**
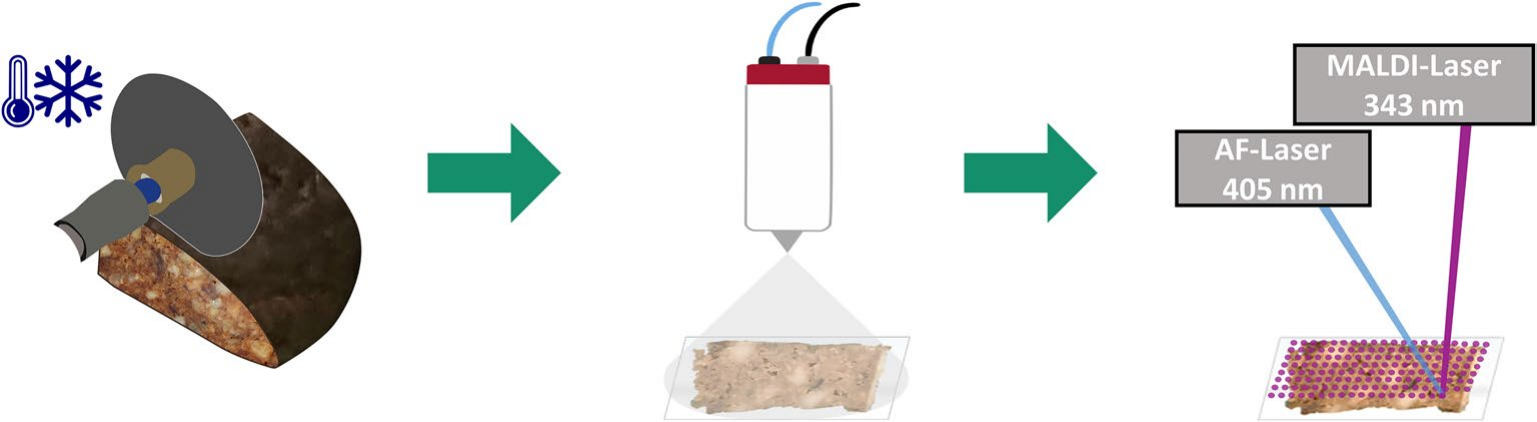
Sample preparation of German gingerbread for MALDI MS imaging experiments. Sections of approx. 2 mm thickness are cut off deep-frozen gingerbread with an electric micro-saw. Sections are glued to an object slide and covered with the MALDI matrix 2.5-dihydroxybenzoic acid (DHB) using an automated sprayer system. The sample is then introduced into an AP-SMALDI5 AF ion source for the autofocusing (AF) MALDI MS imaging measurement. The AF-laser at λ = 405 nm ensures a constant focus of the MALDI-laser throughout the uneven sample surface.

**Figure 2:**
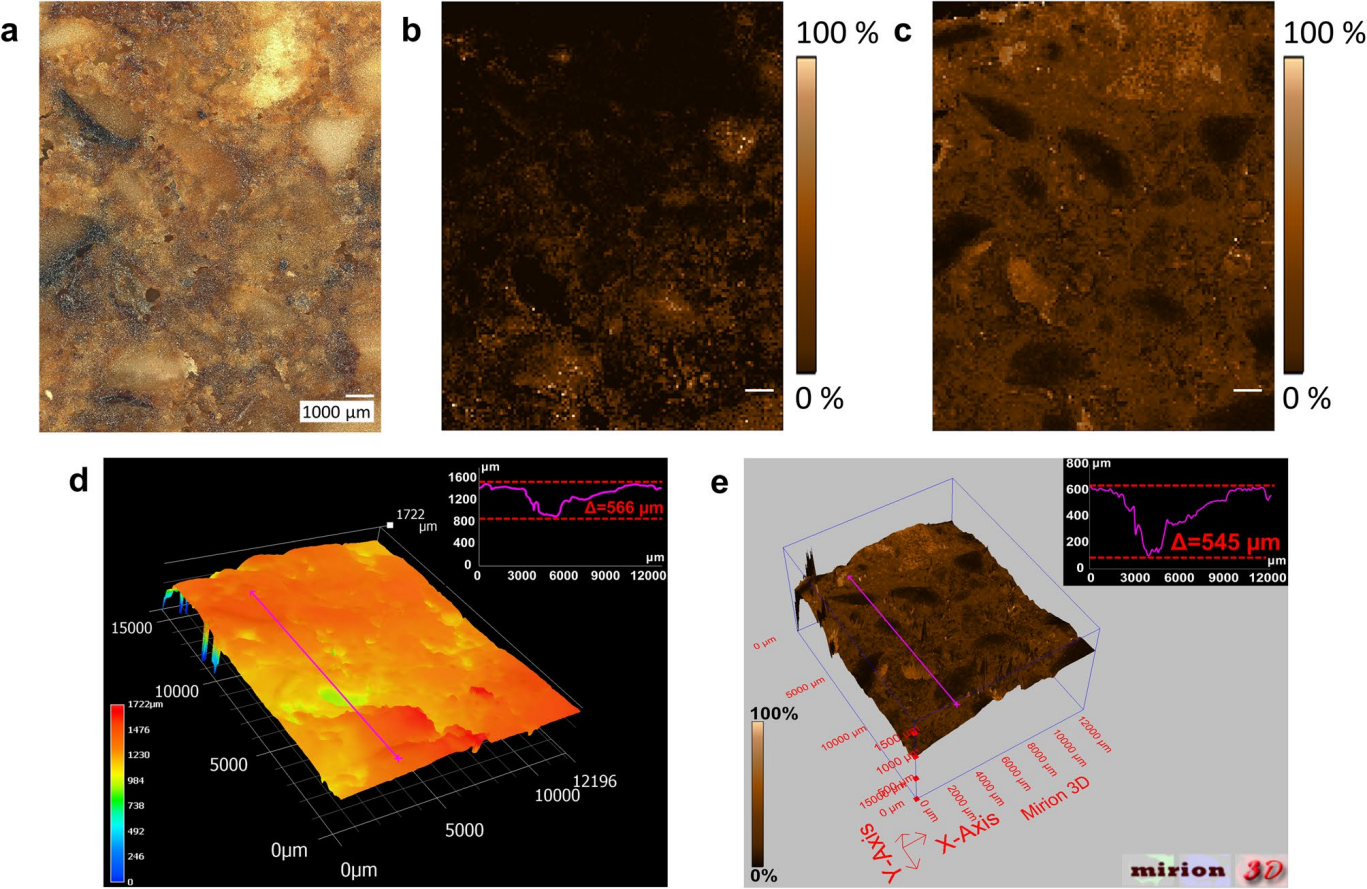
Autofocusing (AF) MALDI MS imaging of German gingerbread. Raster size: 128 × 161 pixels, pixel size: 100 µm (**a**) microscope image of German gingerbread section. (**b**) MS image of PC 36:3 [M + K]^+^, *m/z* 822.54096, TIC-normalized. (**c**) MS image of disaccharide [M + K]^+^, *m/z* 381.07937, TIC-normalized. (**d**) microscopic 3D depth-composition optical overlay with integrated linescan, Z axis is based on microscopic data. (**e**) 3D MS image of disaccharide [M + K]^+^, *m/z* 381.07937, TIC-normalized, Z-axis is based on AF-laser focus data. Gray-scale MS images of the presented analytes and higher saccharides are provided in supplementary, Fig. S9.

The sample preparation workflow for MS imaging of German gingerbread is illustrated in Fig. 1 and described in detail in the following. Highly processed food with soft and/or brittle texture, especially when consisting of multiple separate ingredients, is not suitable for the cryosectioning process^31^. German gingerbread is a typical example belonging to this group of challenging processed food. We recently developed an alternative sectioning method using an electric micro-saw^44^, which was further optimized in the present study. The entire sample was frozen at -80 °C, resulting in a firm structure less prone to deformation. Cutting disks of the micro-saw were thoroughly dried and cooled down to -80 °C prior to sectioning. It has been proven best to keep both the sample and the cutting disks under dry ice at all times except for the immediate sectioning and to use a fresh cutting disk for each section. With this approach, sections of approx. 2 mm thickness could be prepared. For both a homogeneous matrix deposition and reproducible spraying conditions, 2.5-dihydroxybenzoic acid (DHB) matrix was applied onto the gingerbread sections using an HTX M5 Sprayer (HTX Technologies, Chapel Hill, USA) as automated spraying device. To cater to the specific needs of the lipid-rich sample surface, the matrix spray was performed under ‘dry’ conditions, i.e., at a relatively high nozzle-temperature of 85 °C. The matrix density resulting from the parameters set for the automatic sprayer is 23.3 µg/mm^2^, which is comparatively high and at least partly due to the rough section surface. Although when conducted by an experienced operator the section surface is visibly flat and even, a certain topography of around 800 µm in z-direction remained unavoidable (see Fig. 2d). However, when measuring in autofocusing mode (AF-mode), the position of the AF-laser in the MALDI-source is adjusted to the z-position of the current pixel to be irradiated; thus, changes in the topography of the sample surface can be compensated so that the MALDI-laser has a constant focus resulting in high quality mass spectra. The energy and focus settings of the MALDI-laser needed to be optimized as well for gingerbread samples. With the improvement of autofocusing, the standard injection time of 500 ms is sufficient for adequate analyte abundance. Setting an overall defocus and an increased C-trap injection time as done in our initial study^44^ is no longer necessary. For gingerbread measurements, the energy density was approx. 7000 µJ/mm^2^ with 50 applied laser pulses per pixel. Lower laser energy settings, however, resulted in a loss of acrylamide signal. Based on the ablation area caused by the MALDI-laser, measurements with a step size of 100 µm were performed in AF-mode compared to 200 µm in regular setup^44^. It is important to note that spatial resolution in this study is not limited by instrumentation. The identical setup is routinely used to perform high-quality MS imaging at 5 µm pixel size, e.g., of drug compounds and lipids in mouse lung tissue^46^. Instead, the pixel size is limited by the very dry and inhomogeneous properties of the gingerbread sample, which lead to low ionization efficiency and higher signal variation. Consequently, the number of compounds detected in this study is lower compared to typical MS imaging experiments of, e.g., mammalian tissue. Nevertheless, the presented modifications to the sample preparation workflow lay the groundwork for successful autofocusing MALDI MS imaging of German gingerbread.

Using this approach, several gingerbread constituents could be detected and visualized with a substantial increase in contrast. Lipids, such as phosphatidylcholines (PC), are expected to be present in lipophilic areas of the sample, such as nut fragments. PC 36:3 [M + K]^+^, i.e., the potassium adduct of a PC comprising 36 carbon atoms with 3 unsaturated bonds^47^, depicted in Fig. 2b, for instance shows distinct hotspots in nut fragment areas visible in the microscope image (Fig. 2a). The distribution of disaccharide potassium adduct, shown in Fig. 2c, indicates nearly ubiquitous presence of sugar throughout the surface with reduced intensities, e.g., within nut fragments and elevated intensities in a piece of candied fruit and a region of clotted sugar near the upper margin of the image (see supplementary, Fig. S3a). The identity of disaccharide [M + K]^+^ could be confirmed by matching MS/MS-spectra with sucrose standard (see supplementary, Fig. S1). In addition, MS images of tri-, tetra- and pentasaccharides (all K adducts) are provided in Fig. S9d–f. The oligosaccharides show consistent distributions with disaccharide, present in candied fruit, clotted sugar and dough.

The topology of the sample surface is depicted in a microscopic 3D optical overlay together with an exemplary line scan across low and high regions on the z-axis (Fig. 2d). A comparison of the 3D MS image of disaccharide [M + K]^+^ (Fig. 2e) to the microscopic 3D optical overlay (Fig. 2d) shows that the overall topology of the surface could be reproduced by the AF-laser. Moreover, an analogous line scan based on AF-laser data (Fig. 2e, inserted diagram) has a similar profile and comparable overall Δz to the microscopy-based line scan (Fig. 2d, inserted diagram). These results demonstrate the applicability of the AF-laser for uneven surfaces of around 800 µm in depth and the success of autofocusing MALDI MS imaging of German gingerbread with the described parameters.

### MS imaging of the process contaminant acrylamide

**Figure 3:**
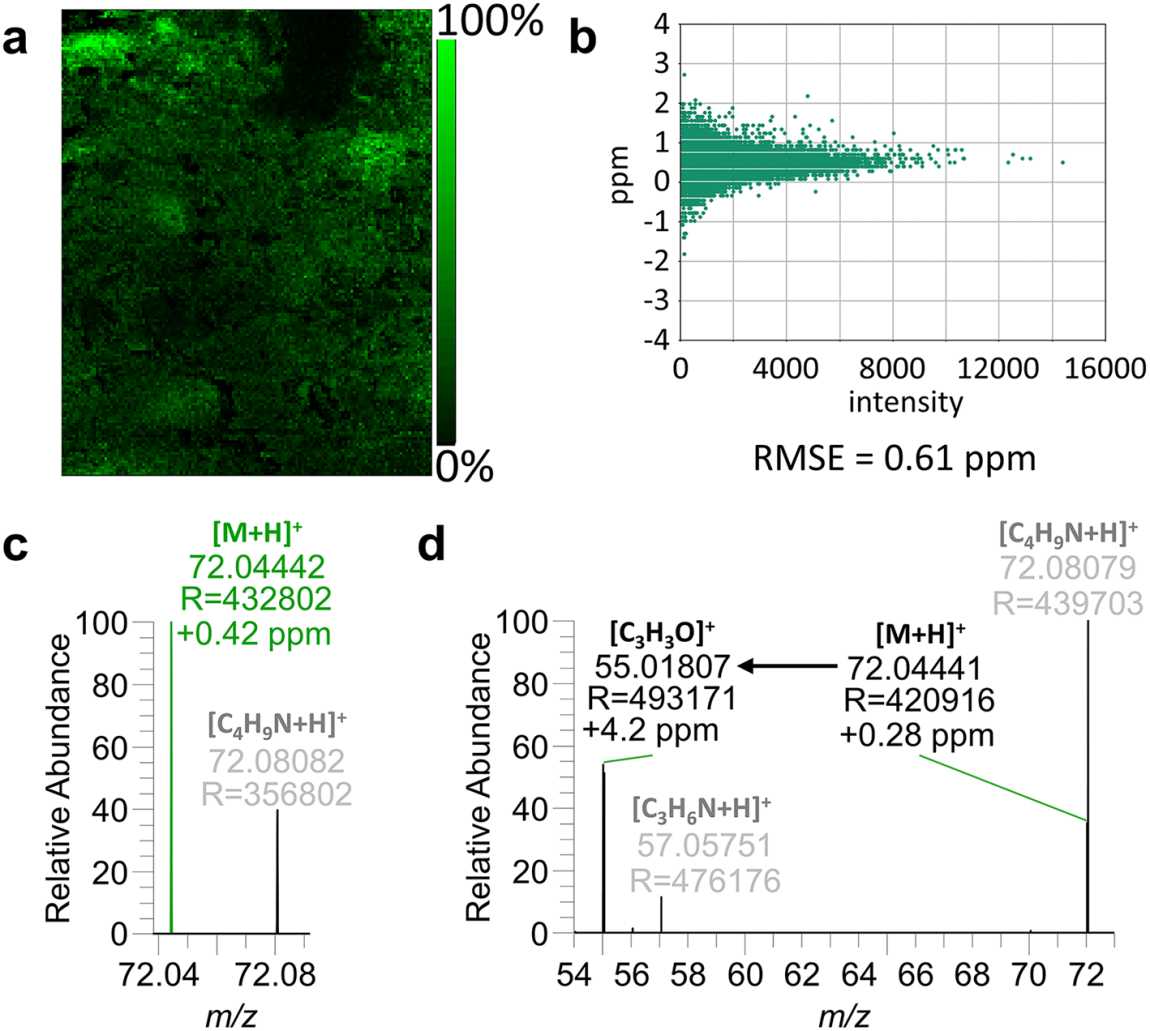
MS imaging of acrylamide in German gingerbread. (**a**) MS image of acrylamide [M + H]^+^, *m/z* 72.04439, TIC-normalized. (**b**) Mass measurement accuracy plot and RMSE value for the acrylamide signal. (**c**) single pixel mass spectrum of acrylamide (**d**) *in-situ* MALDI-MS/MS spectrum showing the characteristic fragment at *m/z* 55.01784, isolation window: *m/z* 72.0 ± 0.2, scan range: *m/z* 50–80, HCD: 60.

In addition to the spatially resolved detection of major constituents, also minor components can be visualized in highly processed food by MALDI MS imaging. In the present example, the distribution of the process contaminant acrylamide is given in Fig. 3a. The average amount of acrylamide in the sample determined by gas chromatography coupled to mass spectrometry (GC–MS) was 3,200 µg/kg (detailed method description is provided in the supplementary material). With a benchmark level of 800 µg/kg set by Commission Regulation (EU) 2017/2158^14^, the sample exceeded this level fourfold. Acrylamide could be detected as its [M + H]^+^ at *m/z* 72.04439 with high mass accuracy as indicated by the mass measurement accuracy (MMA) plot shown in Fig. 3b. Across all 20,363 pixels, in which acrylamide signal could be detected, the root mean square mass error (RMSE) was 0.61 ppm. In case of acrylamide, high mass resolution is crucial due to a neighboring peak at *m/z* 72.08082, as depicted in the single pixel mass spectrum in Fig. 3c. These results could be reproduced in an additional section from the same sample (see supplementary, Fig. S4a–d). The assignment of acrylamide is supported by MALDI-MS/MS experiments on gingerbread surface (Fig. 3d) and of acrylamide standard mixed with DHB matrix on glass (see supplementary, Fig. S2) Both MS/MS spectra show the characteristic fragment signal at its theoretical *m/z* 55.01784 corresponding to the loss of NH_3_. In the MS image shown in Fig. 3a, acrylamide signal is detected in all regions across the section except for the sugar bulk on the top edge of the measurement area and within regions of candied fruit. Judging from the visible intensity distribution, some areas putatively corresponding to nut fragments seem to show higher acrylamide intensities. This observation is addressed in more detail below.

**Figure 4:**
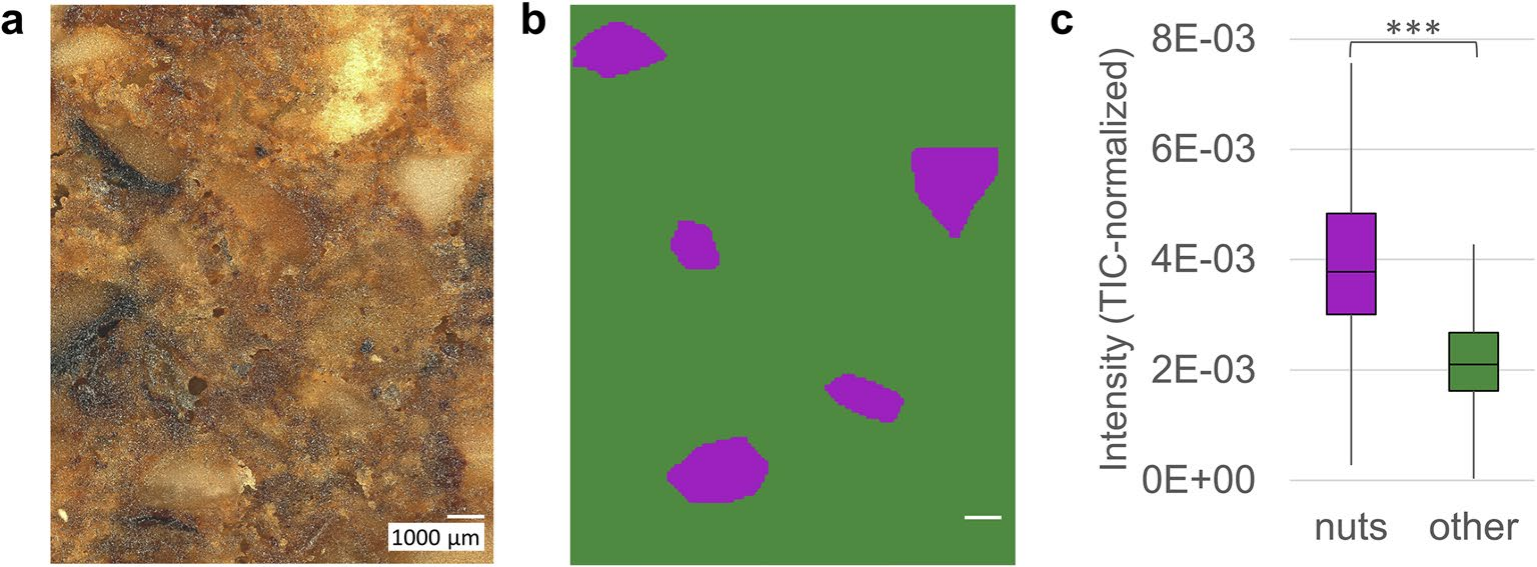
Acrylamide intensity distribution on German gingerbread. (**a**) microscope image of German gingerbread section. (**b**) Surface stratification into regions of interest (ROI) “nuts” based on sum image of triglycerides TG 52:3, TG 52:4, TG 54:5, TG 54:6 and TG 57:10 and “other”; more details are provided in Fig. S3. Export of abundance data of acrylamide *m/z* 72.04439 ± 2.5 ppm for both ROI. (**c**) Boxplot of backtransformed intensity values stratified into ROI “nuts” and “other”; two-sided t-Test was performed on x^0.25^-transformed intensity values; *** t(247) = 16.93, *P* < .001.

In MS imaging with a fixed MALDI-laser focus, uneven surfaces influence the ionization efficiency during the MALDI process. We reduced this effect by using a larger laser ablation area (defocused) in our previous study^44^. By autofocusing prior to each pixel, it is now possible to perform MS imaging with a consistently focused MALDI-laser and, given an adjusted laser energy density, sufficient analyte intensities can still be obtained despite smaller areas of ionized sample compared to defocused measurements. The presented measurements were conducted with a step size of 100 µm without oversampling, i.e., with a MALDI-laser ablation diameter smaller than the step size, thus avoiding multiple irradiations of a given area. Considering the sample size of approx. 12 × 15 mm, this lateral resolution enables more detailed and reliable analyses of analyte distributions across different ingredients compared to previous measurements with a fixed MALDI-laser and 200 µm step size^44^. To support the visible tendency towards higher acrylamide abundances in nut regions, the measurement area was stratified into regions of interest (ROI) comprising distinct nut fragments and an inverted ROI for the remaining sample surface. However, this surface stratification could not simply be based on the optical image since some ingredients, such as sugar agglomerates, might have a similar appearance to nut fragments. Therefore, an evaluation of the optical image was combined with MS imaging information resulting from triglyceride signals typical for nuts. The step-by-step procedure for stratification of gingerbread surface into the ROI “nuts” and “other” is visualized in supplementary, Fig. S3. First, candidate “nut” regions were determined based on visual perception in the optical image (Fig. 4a). In a second step, only regions showing elevated intensities in a sum image of the triglycerides TG 52:3, TG 52:4, TG 54:5, TG 54:6 and TG 57:10 (all [M + K]^+^) were retained, whereas the remaining candidate regions were discarded. The so defined “nut” regions across the sample surface were compiled and exported as one ROI “nuts”; the inverted ROI, i.e., all pixels not assigned to the “nuts” ROI, were exported as “other”. A binary image showing the masks for both ROI is given in Fig. 4b. From these ROI, the acrylamide [M + H]^+^ TIC-normalized intensity values of each pixel were exported as separate datasets. In Fig. 4c, boxplots of the “nuts” and “other” ROI are depicted showing higher acrylamide intensities within the nuts. This finding is supported by a two-sided t-test of the x^0.25^-transformed datasets, proving significantly higher acrylamide intensities within the “nuts” ROI than in the remaining section (*P* < 0.001). Data transformation was necessary to obtain Gaussian distributions within the datasets. In the replicate measurement of the contaminated gingerbread, shown in supplementary, Fig. S4, this protocol was applied as well leading to an analogous result. The higher acrylamide intensities in nut regions may have several possible causes. First, there is a lipid pathway for the formation of acrylamide, which is directly linked to the unsaturation of fatty acids^49^. As previously mentioned and demonstrated in supplementary, Fig. S10, polyunsaturated fatty acids are more abundant in nut regions. Our MS imaging approach allows direct coregistration to the acrylamide distribution in the same measurement, depicted in Fig. 3a, suggesting that this compound class could foster the formation of acrylamide. Secondly, the combination of reduced water activity and a higher heat capacity within nut fragments could further contribute to acrylamide formation. Since acrylamide is formed preferably at low water activity^50^, nut regions might present suitable conditions due to their high fat- and comparatively low water-content. The present results indicate that acrylamide might form preferably, however not exclusively, within regions in and around nut fragments. These findings are in accordance with our previously published two-dimensional MALDI MS imaging of German gingerbread^44^. High acrylamide concentrations of 885 µg/kg in average have been found previously in roasted almonds, whereas roasted hazelnuts contained much less acrylamide^51^. For the sake of producing safe food, it seems likely that not only the size of nut fragments, but also the type and size during roasting are crucial regarding the mitigation of acrylamide in gingerbread^52^. As a further step, a section of German gingerbread with relatively low acrylamide levels (174 µg/kg, determined by GC–MS) has been measured. Images and MMA-plot of the market sample are shown in supplementary, Fig. S5a–c. A comparison of the acrylamide intensities in the presented measurements, depicted in supplementary, Fig. S5d, shows a lower intensity range in the analysis of the market sample compared to the analyses of the contaminated sample and thus proves the applicability of MALDI MS imaging for the analysis not only of heavily contaminated samples, but also of regular market samples. Therefore, the presented protocol can be used as a complementary method to established chromatography-based methods for the analysis of acrylamide in food to gain further insight into the formation process of acrylamide, which is of interest for food safety authorities as well as the food industry.

### Quality control aspects in MS imaging of acrylamide

As described in the previous section, the identity of the detected acrylamide signal has been confirmed by an *in-situ* MALDI-MS/MS-experiment. However, a confirmation of the origin of acrylamide from the contaminated sample itself and not being an artifact resulting from sample preparation or measurement cannot be covered by MS/MS alone. Since acrylamide forms at elevated temperatures, the critical steps in this protocol are sample sectioning and the MALDI-process. Here we present strategies for both steps to exclude the risk of detecting artificially formed acrylamide.

By thorough cooling of both the gingerbread sample and the cutting disk to -80 °C, acrylamide formation resulting from elevated temperatures during the sectioning process has already been addressed. For verification, a measurement of the same sample was sectioned in frozen state with a sharp knife. This method induces no significant heat during the sectioning process and thus artificial production of acrylamide can be excluded. However, sectioning with a knife produces a rougher surface and more cracks. Nonetheless, acrylamide signal could be detected throughout this sample as well with high mass accuracy, as demonstrated in supplementary, Fig. S6. This confirms the presence of acrylamide independent of the sectioning method and excludes artifacts by sample preparation.

Another potential source for acrylamide is the MALDI-process in which the laser deposits energy and thus leads to local increase of temperature at the site of desorption. To confirm that the detected acrylamide signal is not an artifact occurring during this process, we conducted a proof-of-principle experiment on *in-situ* derivatization of acrylamide. Contrary to most previously published studies using derivatization for MS imaging^41–43,53^, the scope is not to enhance the detection limit, but to detect acrylamide on food surfaces with high selectivity. A similar approach has been used for minor bioactive lipids in mouse brain^54^.

**Figure 5:**
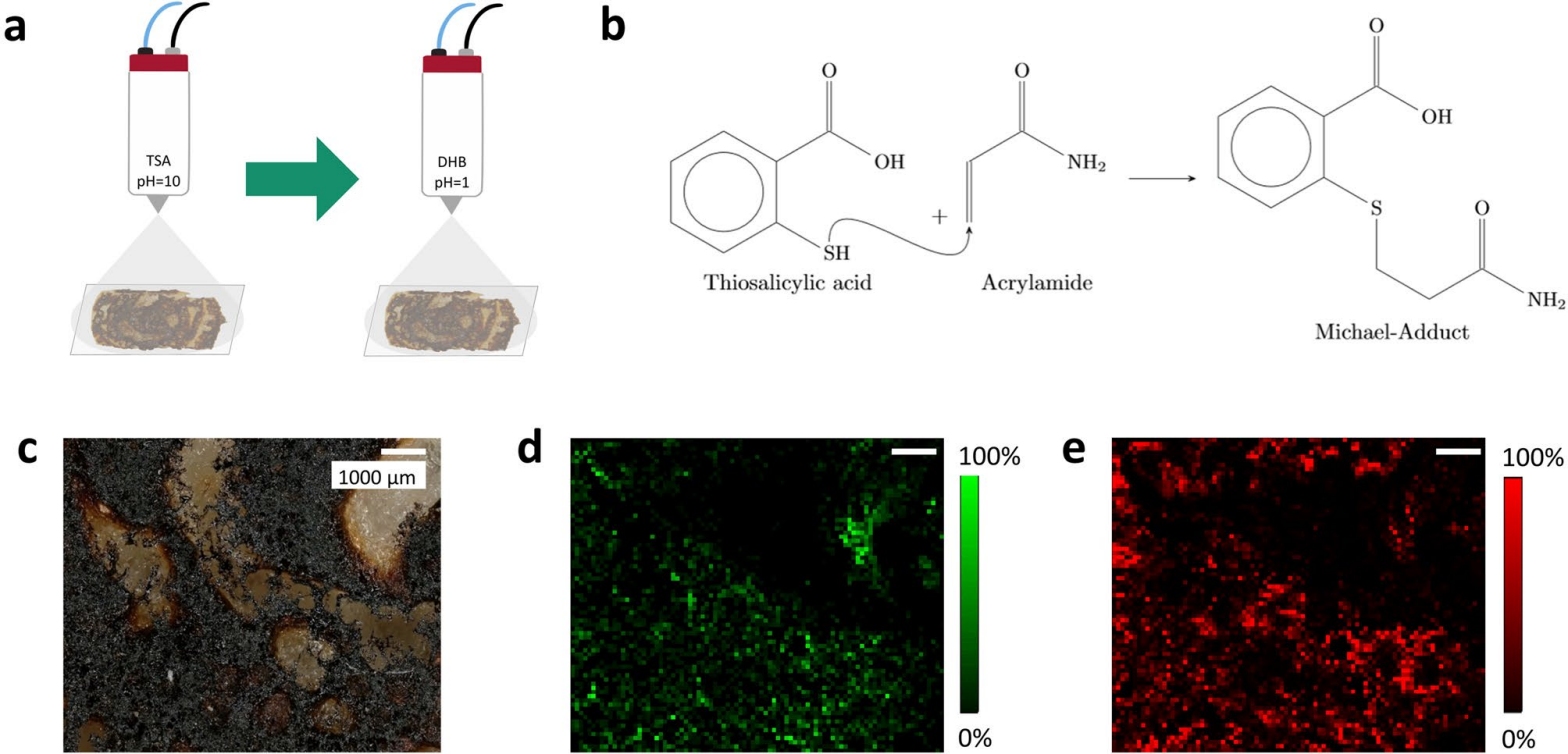
*in-situ* Derivatization of acrylamide on pancake surface. Raster size: 89 × 75 pixels, pixel size: 100 µm. (**a**) Scheme of the sample preparation: Application of thiosalicylic acid in alkaline solution followed by 2.5-dihydroxybenzoic acid in acidic solution. (**b**) Derivatization reaction of thiosalicylic acid and acrylamide. (**c**) Microscope image of the measured pancake surface. (**d**) MS image of acrylamide [M + H]^+^, *m/z* 72.04439. (**e**) MS image of the Michael-adduct [M + Na]^+^, *m/z* 248.03519 (isotope distribution, see supplementary, Fig. S8).

TSA was chosen as derivatization agent for acrylamide based on an established derivatization strategy developed for LC–MS/MS analysis^39^. Alkaline conditions are necessary for an efficient addition of the nucleophilic thiol group to the α,β-unsaturated carbonyl function of acrylamide (see Fig. 5b). Therefore, TSA could not directly be prepared in aqueous methanol, but needed to be dissolved in aqueous NaOH at pH = 10 first and subsequently mixed 50/50 (v/v) with methanol. The derivatization solution was sprayed onto the sample surface using an automated HTX M5 Sprayer. A surface density of 10.3 µg/mm^2^, calculated from the spray parameters, turned out to be the best balance to ensure efficient derivatization and at the same time avoid signal suppression. In contrast to TSA, the MALDI matrix DHB was dissolved in aqueous trifluoroacetic acid (TFA) at pH = 1 and made up with methanol (final mixture 50:50, v/v) to provide an acidic environment on the sample surface and thereby ensure an efficient ionization for MS experiments in positive ion mode. Since excessive crystallized TSA on the sample surface exerts an additional matrix effect by absorbing laser energy, the DHB matrix density was reduced compared to underivatized samples to 16.8 µg/mm^2^. Given the two successive sprays under varying pH-conditions (Fig. 5a), acrylamide derivatization for MALDI MS imaging is not trivial, but needed to be adjusted to meet the respective requirements of the derivatization reagent and an efficient MALDI-process. The laser energy density was reduced to approx. 460 µJ/mm^2^ to avoid fragmentation of the labile derivatization product.

In our gingerbread samples, acrylamide levels were sufficient for the detection of its protonated ion, but too low for the derivatization product to be detected (see supplementary, Fig. S7). Consequently, we used freshly prepared pancakes as a model for acrylamide-containing food matrix to demonstrate the feasibility of the derivatization method. In Fig. 5c, an optical image of the measured pancake surface is presented. The acrylamide [M + H]^+^ signal at *m/z* 72.04439 was detected analogous to gingerbread samples with an RMSE = 0.57 ppm (2596 spectra), the corresponding MS image is provided in Fig. 5d. Acrylamide is distributed throughout vast parts of pancake surface. With a similar distribution, the TSA-acrylamide adduct [M + Na]^+^ at *m/z* 248.03519 could be detected on pancake surface (Fig. 5e). To confirm the identity of the sodiated TSA-acrylamide adduct, mass spectra showing ^13^C- and ^34^S-isotope distributions of both the adduct formed from acrylamide standard on glass and from pancake surface are shown in supplementary, Fig. S8.

The detection of TSA-acrylamide adduct confirms that the detected acrylamide originates from the sample itself and is not a result of the ionization process. In addition, this derivatization approach covers the entire sample area, in contrast to commonly conducted *in-situ* MALDI-MS/MS experiments to confirm the identity of analytes, which are typically limited to a small representative area on the measured surface.

## Conclusions

MALDI MS imaging of German gingerbread as an example of processed food has been optimized by introducing autofocusing of the MALDI-laser to adjust for height differences of the sample with an uneven surface. The contaminant acrylamide was successfully detected and identified in sections of German gingerbread by means of accurate mass and MS/MS measurements. A comparison of acrylamide intensities in different sample compartments revealed significantly higher intensities in regions containing nuts. In addition, by identifying critical steps for unintended acrylamide formation and complementary measurements including a new proof-of-principle approach for *in-situ* derivatization of acrylamide, we present measures to reduce the risk of artifacts during sample preparation and the MALDI-process.

## Materials and methods

### Sample material

Traditional German gingerbread was provided by the Bavarian Health and Food Safety Authority (LGL). The sample contained 3,200 µg/kg acrylamide previously determined by GC–MS in official analyses (for the method description, see supplementary material). Furthermore, a reference sample of traditional German gingerbread was purchased on a local market.

For the investigation of derivatized acrylamide, pancakes were prepared from a mixture of wheat flour, glucose and water with added asparagine (1.5%m). The dough was baked in a pan without using oil until a dark-brown surface emerged.

### Chemicals and standards

HPLC-Grade methanol and water were purchased from Carl Roth (Karlsruhe, Germany). 2,5-Dihydroxybenzoic acid (DHB), 2-Sulfanylbenzoic acid (thiosalicylic acid, TSA), sucrose and acrylamide analytical standard were purchased from Sigma Aldrich (Dreieich, Germany). Adhesive glass slides (Menzel Gläser, SuperFrost) were purchased from VWR (Darmstadt, Germany).

### Sample sectioning

Whole gingerbreads were first cut into quarters using a sharp knife and then frozen at -80 °C. If applicable, the chocolate coating was removed from the frozen sample with a knife prior to sectioning. Sections of 2–3 mm thickness were acquired using an electric micro saw (Dremel 8220, Breda, Netherlands). Gingerbread sections were always freshly prepared at the day of analysis.

### TSA application

Thiosalicylic acid (TSA) was applied using an HTX M5 Sprayer (HTX Technologies, Chapel Hill, USA). The spray solution was prepared as follows: 154 mg TSA were weighed into a glass beaker and suspended in 3 mL water. By adding 1 M NaOH, the suspension was brought to pH = 10 and then filled up to 5 mL. Under these alkaline conditions, TSA is completely dissolved. The solution is finally made up to 10 mL with methanol and loaded into the sample loop of the sprayer. 20 passes were sprayed onto the sample surface with a flow rate of 50 µL/min, a spray nozzle velocity of 750 mm/min and 50 °C nozzle temperature. Track spacing was set to 2 mm in a criss-cross (CC) scan-pattern.

### Matrix application

For the application of DHB matrix, the HTX M5 Sprayer was used as well. The matrix solution was prepared in methanol/water (1:1, (v/v)) to a final concentration of 40 mg/mL and 30 mg/mL for gingerbread and pancake samples, respectively, and acidified with 0.4 % TFA. For gingerbread samples, 14 passes were sprayed onto the sample surface with a flow rate of 100 µL/min, a spray nozzle velocity of 1200 mm/min and 85 °C nozzle temperature. For the pancake samples, the DHB solution was sprayed onto the sample directly after the derivatization using the same spraying system with minimal time delay due to a manual flushing step of the system. 16 passes were sprayed with a flow rate of 70 µL/min, a velocity of 1000 mm/min and 80 °C nozzle temperature. Track spacing was always set to 2 mm in a criss-cross (CC) scan-pattern.

### AF-MALDI MS imaging measurements

All MALDI MS imaging measurements were conducted using a quadrupole-orbitrap mass spectrometer QExactive HF (Thermo Fisher Scientific GmbH, Bremen, Germany) coupled to the atmospheric pressure autofocusing MALDI imaging source AP-SMALDI5 AF (TransMIT GmbH, Gießen, Germany)^45^. Apart from the diode-pumped solid-state laser (MALDI-laser, λ = 343 nm) operating at a repetition rate of 100 Hz and oriented orthogonally to the sample surface, the ion source is equipped with a triangulation laser (AF-laser, λ = 405 nm), which is directed at the same spot as the MALDI laser, but from a 35° angle relative to the MS transfer capillary axis^45^. All measurements were carried out in positive ion mode with a fixed C-trap injection time of 500 ms and 50 laser shots per pixel at a mass resolution of 240,000 @ *m/z* 200 full width at half maximum (FWHM). To ensure high mass accuracy (< 1.5 ppm RMSE), an online mass calibration before and internal mass calibration during each measurement were performed using homogeneously distributed matrix cluster ions^55^. On gingerbread samples, the two *m/z* ranges 70–280 and 250–1000 were scanned successively and with shifted x- and y-coordinates (0.5 × step size) to cover both the mass range of small (e.g., acrylamide) and larger analytes, such as sugars and lipids. For the pancake samples, only one measurement with an *m/z* range 70–280 was conducted.

### Data analysis

Optical images and depth composition heatmaps of the samples were created using a Keyence VHX-5000 digital microscope (Keyence Corporation, Osaka, Japan). Analysis of MS and MS/MS data was conducted in QualBrowser, a tool within the Thermo Xcalibur v4.0.27.10 software package. Following MS imaging experiments, proprietary Thermo “RAW” files were converted to imzML using RAW2IMZML converter v1.3R85 (TransMIT GmbH, Gießen, Germany) and validated with imzML Validator v1.0.2^56^. 2D and 3D ion images were generated based on a bin width of ± 3 ppm using Mirion 3D v3.3.64.11^57^. For the visualization of gingerbread ingredients, a customized “ginger” colormap was created (available for download, see supplementary information). SpectralAnalysis was used for the creation and export of regions of interest (ROI)^58^; whereas the export of acrylamide abundance data was performed in MSiReader v1.01x^59^. The generation of MMA plots and RMSE calculation were conducted using an in-house programmed imzML Analyzer v2.3.3 software. Statistical analyses were performed in IBM SPSS Statistics v26.0.0.0. The level of significance was defined as *P* ≤ 0.05.

## Supplementary Information

Supplementary Information 1.

Supplementary Information 2.

## Data Availability

Further data are available from the corresponding author on request.

## References

[1] Tareke E, Rydberg P, Karlsson P, Eriksson S, Törnqvist M (2002). Analysis of acrylamide, a carcinogen formed in heated foodstuffs. J. Agric. Food Chem..

[2] Mottram DS, Wedzicha BL, Dodson AT (2002). Acrylamide is formed in the Maillard reaction. Nature.

[3] Stadler RH (2002). Acrylamide from Maillard reaction products. Nature.

[4] Besaratinia A, Pfeifer GP (2004). Genotoxicity of acrylamide and glycidamide. J. Natl. Cancer Inst..

[5] Calleman, C. J., Bergmark, E. & Costa, L. Acrylamide is metabolized to glycidamide in the rat: evidence from hemoglobin adduct formation. 10.1021/tx00017a004 2133091

[6] International Agency for Research on Cancer. *Some Industrial Chemicals: IARC Monographs on the Evaluation of Carcinogenic Risks to Humans*. 560 (1994). PMC7681540 7869568

[7] *Annex VI Part 3 Table 3.1 Regulation (EC) 1232/2008 of the European Parliament and of the Council of 16 December 2008 on classification, labelling and packaging of substances and mixtures, amending and repealing Directives 67/548/EEC and 1999/45/EC, and amending Regulation (EC) 1907/2006*

[8] Cantrell MS, McDougal OM (2021). Biomedical rationale for acrylamide regulation and methods of detection. Compr. Rev. Food Sci. Food Saf..

[9] Chain EPOCITF (2015). Scientific opinion on acrylamide in food. EFSA J..

[10] Goempel K (2017). Biomarker monitoring of controlled dietary acrylamide exposure indicates consistent human endogenous background. Arch. Toxicol..

[11] Eisenbrand G (2020). Revisiting the evidence for genotoxicity of acrylamide (AA), key to risk assessment of dietary AA exposure. Arch. Toxicol..

[12] Joint FAO/WHO Expert Committee on Food Additives (JECFA). *CODE OF PRACTICE FOR THE REDUCTION OF ACRYLAMIDE IN FOODS*. (CAC/RCP 67-2009), 11 (2009). https://www.fao.org/fao-who-codexalimentarius/sh-proxy/en/?lnk=1&url=https%253A%252F%252Fworkspace.fao.org%252Fsites%252Fcodex%252FStandards%252FCAC%2BRCP%2B67-2009%252FCXP_067e.pdf.

[13] Food & Drug Administration. *Guidance for Industry: Acrylamide in Foods*. (FDA-2013-D-0715), (2013). https://www.fda.gov/regulatory-information/search-fda-guidance-documents/guidance-industry-acrylamide-foods.

[14] *Commission Regulation (EU) 2017/2158 of 20 November 2017 establishing mitigation measures and benchmark levels for the reduction of the presence of acrylamide in food*

[15] Hoenicke K, Gatermann R (2005). Studies on the stability of acrylamide in food during storage. J. AOAC Int..

[16] Arvanitoyannis IS, Dionisopoulou N (2014). Acrylamide: formation, occurrence in food products, detection methods, and legislation. Crit. Rev. Food Sci. Nutr..

[17] *Art. 1 (1) in conjunction with Annex IV Regulation (EU) 2017/2158*

[18] *Leitsätze für Feine Backwaren vom 17./18. September 1991 (Beilage Nr. 86b zum BAnz. Vom 8. Mai 1992, GMBl. Nr. 17 S. 325 vom 8. Mai 1992), zuletzt geändert am 09.01.2010 (BAnz. Nr. 16 vom 29.01.2010, GMBl. Nr. 5/6 S. 120 ff. vom 04.02.2010)*

[19] *Recital 3 in conjunction with Annex Commission Recommendation (EU) 2019/1888 of 7 November 2019 on the monitoring of the presence of acrylamide in certain foods*

[20] Mousavi KA, Fakhri Y, Nematollahi A, Seilani F, Vasseghian Y (2020). The concentration of acrylamide in different food products: A global systematic review, meta-analysis, and meta-regression. Food Rev. Int..

[21] Ščupáková K (2020). Cellular resolution in clinical MALDI mass spectrometry imaging: the latest advancements and current challenges. Clin. Chem. Lab. Med..

[22] Bhandari DR (2015). High resolution mass spectrometry imaging of plant tissues: towards a plant metabolite atlas. Analyst.

[23] Neumann, E. K., Do, T. D., Comi, T. J. & Sweedler, J. V. Exploring the fundamental structures of life: non-targeted, chemical analysis of single cells and subcellular structures. *Angewandte Chemie (International ed. in English)* **58**, 9348–9364. 10.1002/anie.201811951 (2019). 10.1002/anie.201811951 PMC6542728 30500998

[24] Schnackenberg LK, Thorn DA, Barnette D, Jones EE (2022). MALDI imaging mass spectrometry: An emerging tool in neurology. Metab. Brain Dis..

[25] Bednarz H, Roloff N, Niehaus K (2019). Mass Spectrometry Imaging of the Spatial and Temporal Localization of Alkaloids in Nightshades. J. Agric. Food Chem..

[26] Hou J (2022). Spatial lipidomics of eight edible nuts by desorption electrospray ionization with ion mobility mass spectrometry imaging. Food Chem..

[27] Da Silva LG (2022). Laser ablation electrospray ionization mass spectrometry imaging as a new tool for accessing patulin diffusion in mold-infected fruits. Food Chem..

[28] Marzec ME, Wojtysiak D, Połtowicz K, Nowak J, Pedrys R (2016). Study of cholesterol and vitamin E levels in broiler meat from different feeding regimens by TOF-SIMS. Biointerphases.

[29] Yukihiro Y, Zaima N (2020). Application of mass spectrometry imaging for visualizing food components. Foods (Basel, Switzerland).

[30] Gallego M, Mora L, Toldrá F (2018). Differences in peptide oxidation between muscles in 12 months Spanish dry-cured ham. Food Res. Int. (Ottawa, Ont.).

[31] Fowble KL, Okuda K, Cody RB, Musah RA (2019). Spatial distributions of furan and 5-hydroxymethylfurfural in unroasted and roasted Coffea arabica beans. Food Res. Int. (Ottawa, Ont.).

[32] Rešetar Maslov D, Svirkova A, Allmaier G, Marchetti-Deschamann M, Kraljević PS (2019). Optimization of MALDI-TOF mass spectrometry imaging for the visualization and comparison of peptide distributions in dry-cured ham muscle fibers. Food Chem..

[33] Boughton BA, Thinagaran D, Sarabia D, Bacic A, Roessner U (2016). Mass spectrometry imaging for plant biology: A review. Phytochem. Rev..

[34] Qi B-L (2014). Derivatization for liquid chromatography-mass spectrometry. TrAC Trends Anal. Chem..

[35] Lim H-H, Shin H-S (2014). A new derivatization approach with D-cysteine for the sensitive and simple analysis of acrylamide in foods by liquid chromatography-tandem mass spectrometry. J. Chromatogr. A.

[36] Yang S (2019). Thiol-ene click derivatization for the determination of acrylamide in potato products by capillary electrophoresis with capacitively coupled contactless conductivity detection. J. Agric. Food Chem..

[37] Martínez E, Rodríguez JA, Bautista M, Rangel-Vargas E, Santos EM (2018). Use of 2-naphthalenethiol for derivatization and determination of acrylamide in potato crisps by high-performance liquid chromatographic with fluorescence detection. Food Anal. Methods.

[38] Faraji M, Hamdamali M, Aryanasab F, Shabanian M (2018). 2-Naphthalenthiol derivatization followed by dispersive liquid-liquid microextraction as an efficient and sensitive method for determination of acrylamide in bread and biscuit samples using high-performance liquid chromatography. J. Chromatogr. A.

[39] Jezussek M, Schieberle P (2003). A new LC/MS-method for the quantitation of acrylamide based on a stable isotope dilution assay and derivatization with 2-mercaptobenzoic acid Comparison with two GC/MS methods. J. Agric. Food Chem..

[40] Oellig C, Gottstein E, Granvogl M (2022). Analysis of acrylamide in vegetable chips after derivatization with 2-mercaptobenzoic acid by liquid chromatography–mass spectrometry. Eur. Food Res. Technol..

[41] Dueñas ME, Larson EA, Lee YJ (2019). Toward mass spectrometry imaging in the metabolomics scale: increasing metabolic coverage through multiple on-tissue chemical modifications. Front. Plant Sci..

[42] Merdas M (2021). On-tissue chemical derivatization reagents for matrix-assisted laser desorption/ionization mass spectrometry imaging. J Mass Spectr.

[43] Harkin C (2021). On-tissue chemical derivatization in mass spectrometry imaging. Mass Spectrom. Rev..

[44] Kokesch-Himmelreich J (2022). MALDI mass spectrometry imaging: from constituents in fresh food to ingredients, contaminants and additives in processed food. Food Chem..

[45] Kompauer M, Heiles S, Spengler B (2017). Autofocusing MALDI mass spectrometry imaging of tissue sections and 3D chemical topography of nonflat surfaces. Nat. Methods.

[46] Kokesch-Himmelreich J (2022). Do anti-tuberculosis drugs reach their target?-high-resolution matrix-assisted laser desorption/ionization mass spectrometry imaging provides information on drug penetration into necrotic granulomas. Anal. Chem..

[47] Liebisch G (2020). Update on LIPID MAPS classification, nomenclature, and shorthand notation for MS-derived lipid structures. J. Lipid. Res..

[48] Buchberger AR, DeLaney K, Johnson J, Li L (2018). Mass spectrometry imaging: A review of emerging advancements and future insights. Anal. Chem..

[49] Daniali G, Jinap S, Hajeb P, Sanny M, Tan CP (2016). Acrylamide formation in vegetable oils and animal fats during heat treatment. Food Chem..

[50] Biedermann M, Biedermann-Brehm S, Noti A, Grob K (2002). Methods for determining the potential of acrylamide formation and its elimination in raw materials for food preparation, such as potatoes. Mitt. Lebensmittelunters. Hyg..

[51] Amrein TM (2005). Acrylamide in roasted almonds and hazelnuts. J. Agric. Food Chem..

[52] Weisshaar, R. Acrylamide in bakery products-results from model experiments. *DEUTSCHE LEBENSMITTEL-RUNDSCHAU* **100**, 92 (2004).

[53] Enomoto H, Sensu T, Yumoto E, Yokota T, Yamane H (2018). Derivatization for detection of abscisic acid and 12-oxo-phytodienoic acid using matrix-assisted laser desorption/ionization imaging mass spectrometry. Rapid Commun. Mass Spectrom. RCM.

[54] Iwama T (2021). Development of an on-tissue derivatization method for MALDI mass spectrometry imaging of bioactive lipids containing phosphate monoester using phos-tag. Anal. Chem..

[55] Treu A, Römpp A (2021). Matrix ions as internal standard for high mass accuracy matrix-assisted laser desorption/ionization mass spectrometry imaging. Rapid Commun. Mass Spectrom..

[56] Race AM, Römpp A (2018). Error-free data visualization and processing through imzML and mzML validation. Anal. Chem..

[57] Paschke C (2013). Mirion–a software package for automatic processing of mass spectrometric images. J. Am. Soc. Mass Spectrom..

[58] Race AM (2016). SpectralAnalysis: Software for the masses. Anal. Chem..

[59] Bokhart MT, Nazari M, Garrard KP, Muddiman DC (2018). MSiReader v1.0: Evolving open-source mass spectrometry imaging software for targeted and untargeted analyses. J. Am. Soc. Mass Spectrom..

