# Supplementary material for: Autofocusing MALDI MS imaging of processed food exemplified by the contaminant acrylamide in German gingerbread
Source: Sci Rep. 2023 Apr 3;13:5400. doi: 10.1038/s41598-023-32004-w (PMC10070467; doi:10.1038/s41598-023-32004-w)
Supplement: Supplementary file 1 — Supplementary Information 1. [file 41598_2023_32004_MOESM1_ESM.pdf]

Autofocusing MALDI MS Imaging of processed food exemplified by the contaminant acrylamide in German gingerbread

Oliver Wittek, Andreas Römpp

Bioanalytical Sciences and Food Analysis, University of Bayreuth, Universitaetsstrasse. 30, D-95440 Bayreuth, Germany

Supplementary

GC-MS reference method for acrylamide quantification

After soaking the sample in warm water, turbids are removed from the extract using Carrez precipitation. The clarified extract is treated with a bromination reagent and concentrated by solid phase extraction (SPE). The derivatized acrylamide is eluted from the column with ethyl acetate, concentrated by evaporation and then analyzed by GC-MS. Quantification is performed via external calibration under consideration of initially added deuterated internal standard acrylamide-d3. The limit of quantification is 33 µg/kg.

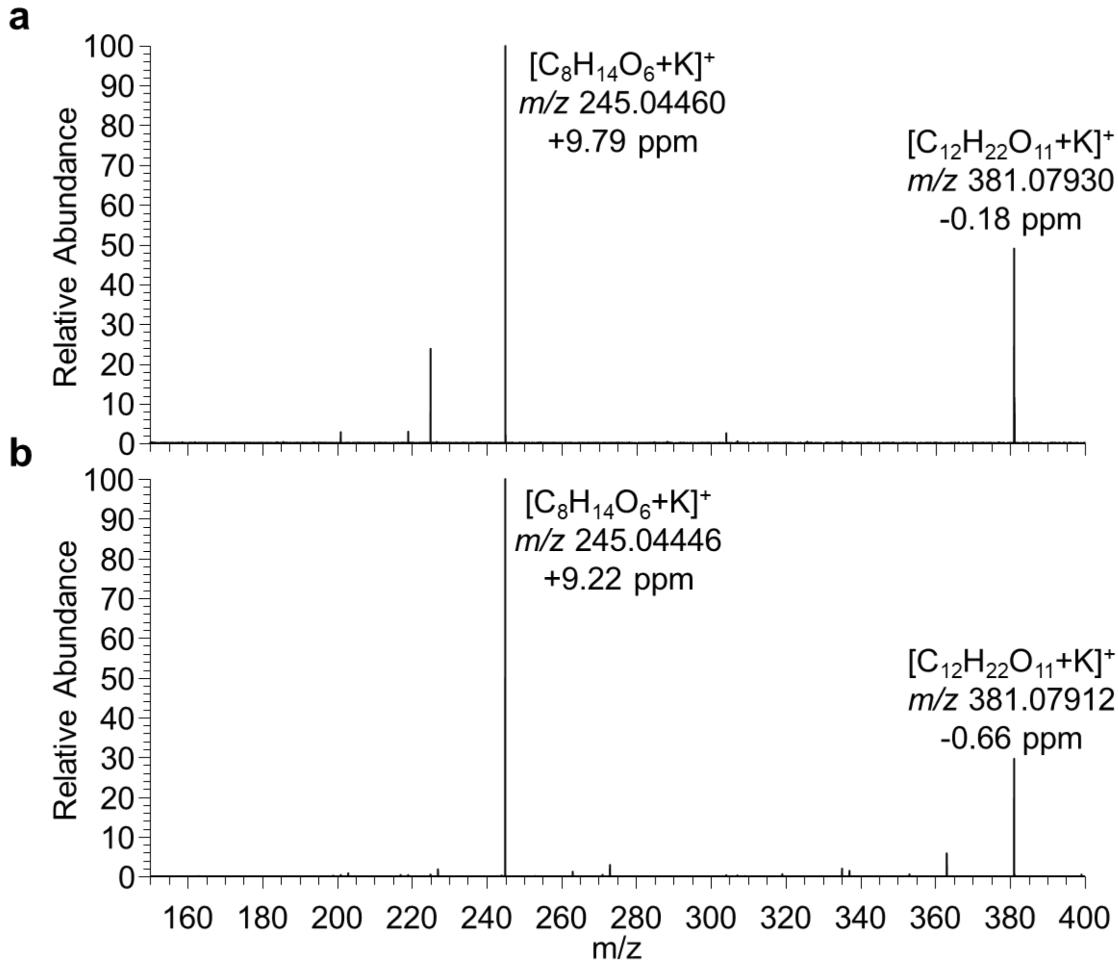

Fig. S1: Average MALDI-MS/MS spectra of (**a**) putative disaccharide $[M+K]^+$ on gingerbread, theoretical *m/z* 381.07937 (n=47 single spectra), isolation window: *m/z* 381.1 ± 0.2 at NCE = 31 and (**b**) sucrose standard $[M+K]^+$ on glass, theoretical *m/z*. 381.07937 (n=84 single spectra), isolation window: *m/z* 381.1 ± 0.2 at NCE = 20.

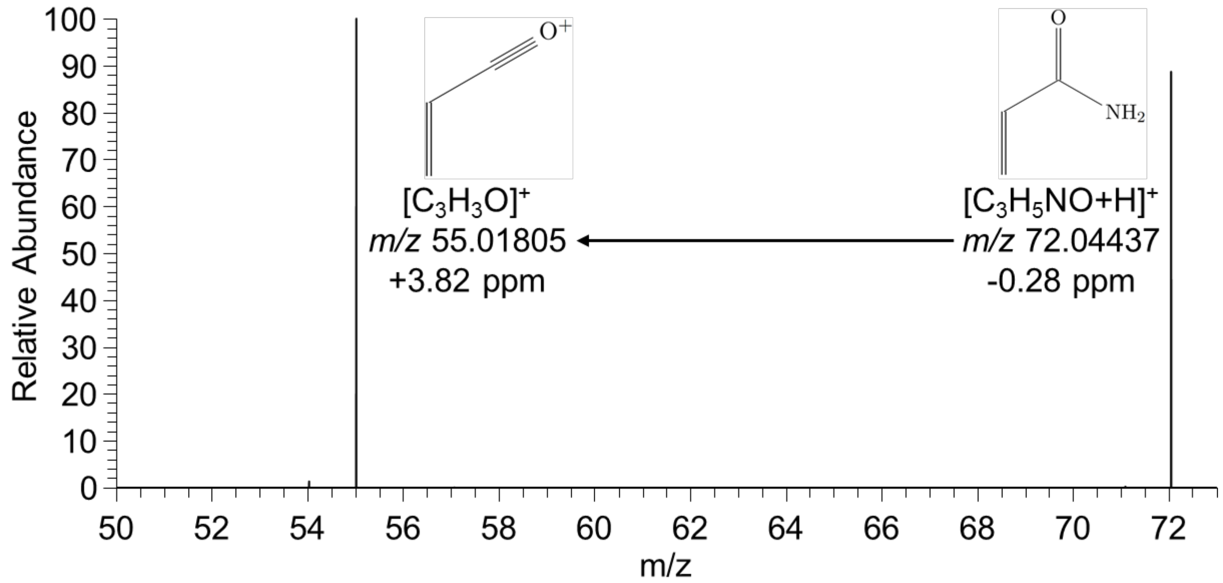

Fig. S2: Average MALDI MS/MS spectrum of acrylamide standard (n=42 single spectra). Isolation window: *m/z* 72.0 ± 0.2 at NCE_{HCD} = 50.

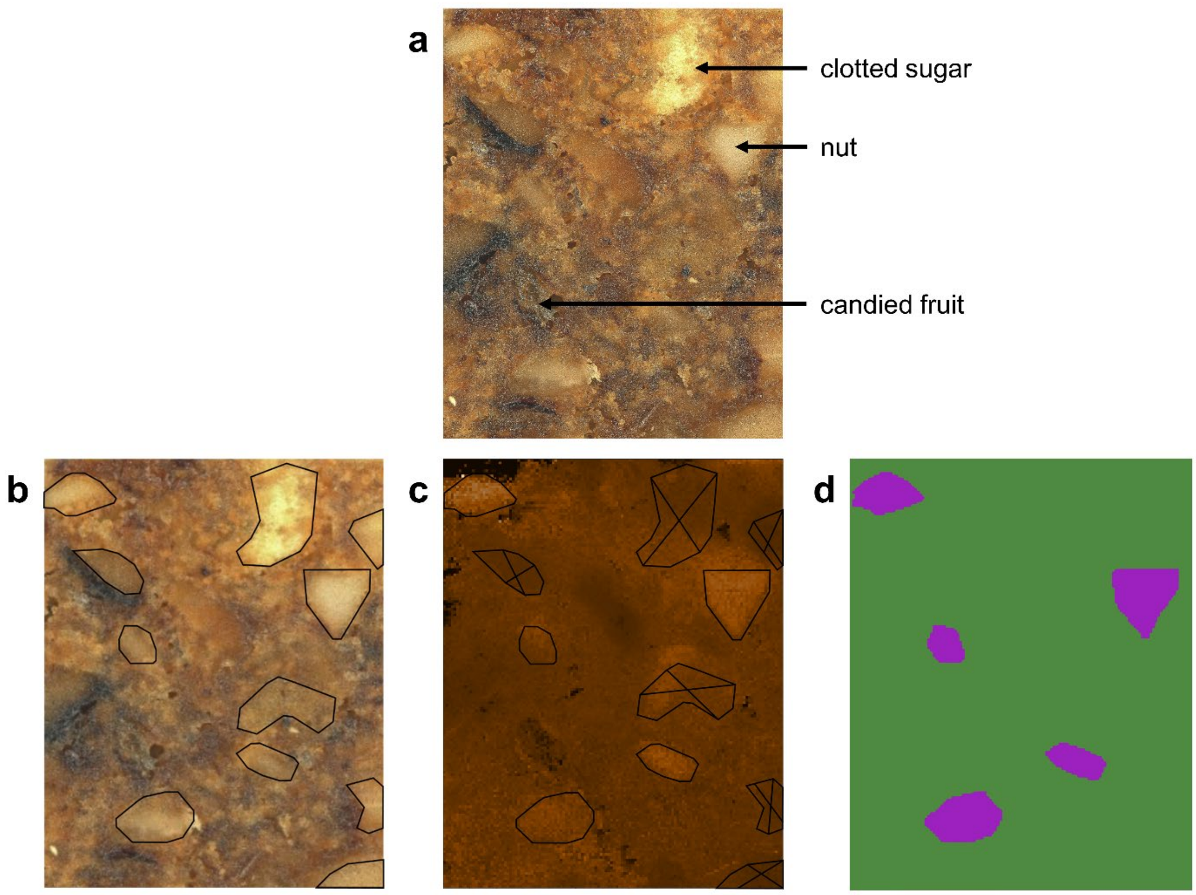

Fig. S3: Step-by-step procedure for stratification of gingerbread surface into ROI "nuts" and "other". (**a**) optical image with annotated ingredients. (**b**) optical image with potential nut fragments based on visual perception. (**c**) sum image of triglycerides TG 52:3, TG 52:4, TG 54:5, TG 54:6 and TG 57:10 (all $[M+K]^+$), generated in Mirion[52], with overlaid candidate "nuts" ROI; regions with no visibly elevated triglyceride intensity were crossed out. (**d**) ROI mask for export of acrylamide abundance data; ROI "nuts" is depicted in pink, ROI "other" in green, performed in SpectralAnalysis[53]. Export of abundance data of acrylamide *m/z* 72.04439 ± 2.5 ppm for both ROI, performed in MSiReader v1.01x[54].

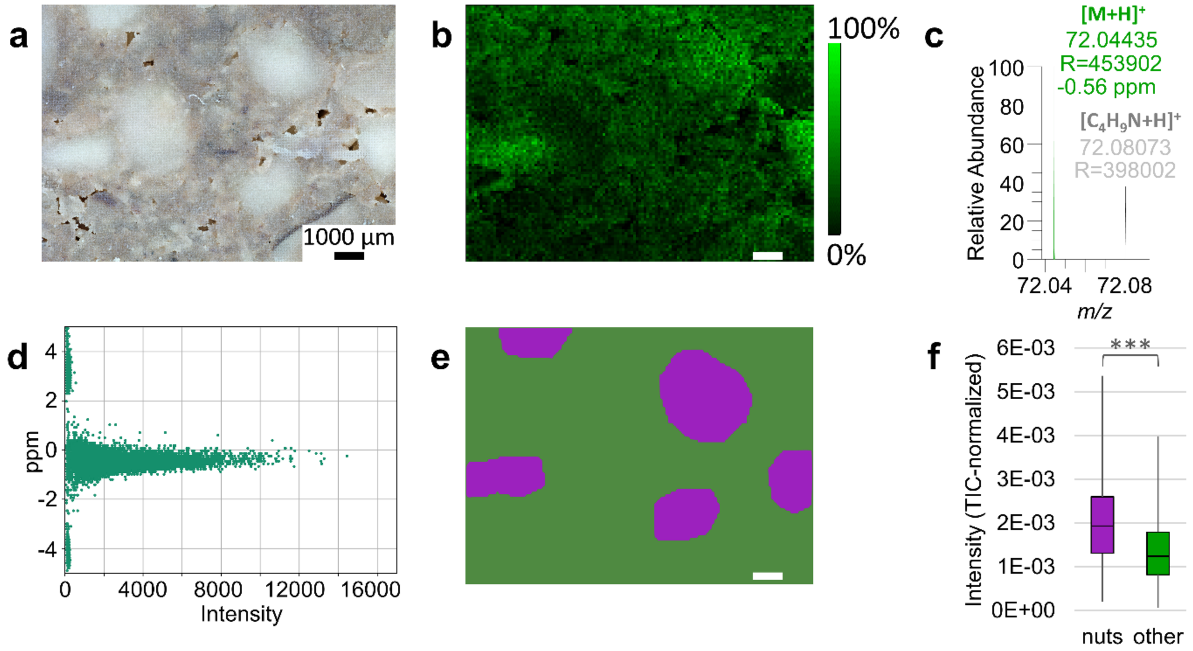

Fig. S4: MS imaging of a replicate contaminated German gingerbread sample. (**a**) optical image after measurement. (**b**) MS image of acrylamide $[M+H]^+$, *m/z* 72.04439, TIC-normalized. Image dimensions are 127x94 pixels with a step size of 100 µm and an *m/z* range of 70-280. (**c**) single pixel mass spectrum of acrylamide signal together with mass resolution R = 453902 and relative mass error of -0.56 ppm. (**d**) mass measurement accuracy plot of *m/z* 72.04439, RMSE = 0.52 ppm (n=11896 peaks). (**e**) binary image showing ROI “nuts”, based on optical image and sum image of triglycerides TG 52:3, TG 52:4, TG 54:5, TG 54:6 and TG 57:10, and “other”. (**f**) Boxplot of backtransformed intensity values stratified into ROI „nuts“ and „other“; two-sided t-Test was performed on $x^{0.5}$-transformed intensity values; *** $t(3087)=32.26$, $p<.001$.

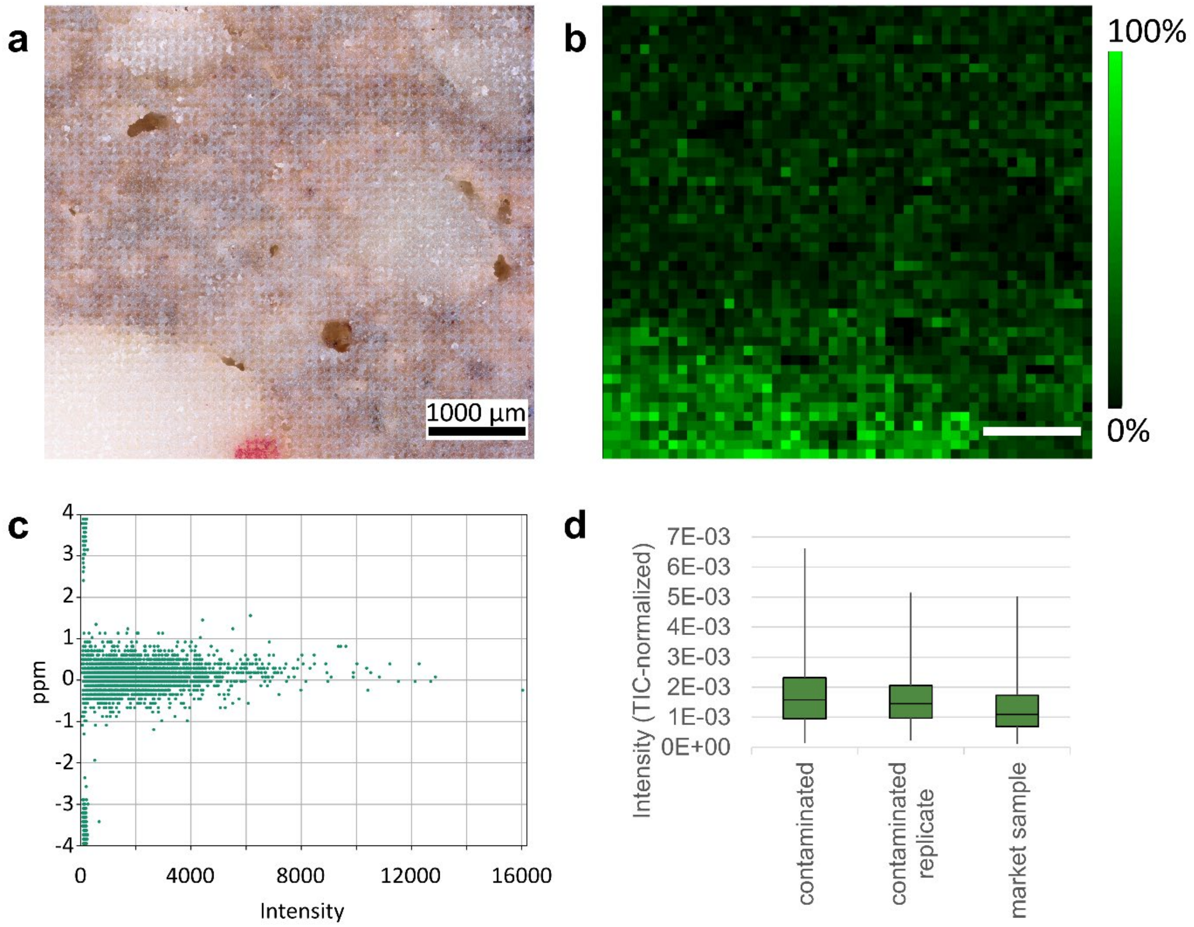

Fig. S5: MS imaging of a gingerbread market sample. The mean acrylamide amount determined by the GC-MS reference method is 174 µg/kg. (**a**) optical image after measurement. (**b**) MS image of acrylamide $[M+H]^+$, *m/z* 72.04439, TIC-normalized. Image dimensions are 52x48 pixels with a step size of 100 µm and an *m/z* range of 70-280. (**c**) mass measurement accuracy plot of *m/z* 72.04439, RMSE = 0.37 ppm (n=2469 peaks). (**d**) Boxplot of backtransformed acrylamide intensity values of entire measurements. Comparison of acrylamide intensities between the contaminated sample, shown in Fig. 3, the replicate measurement, shown in Fig. S8, and the market sample.

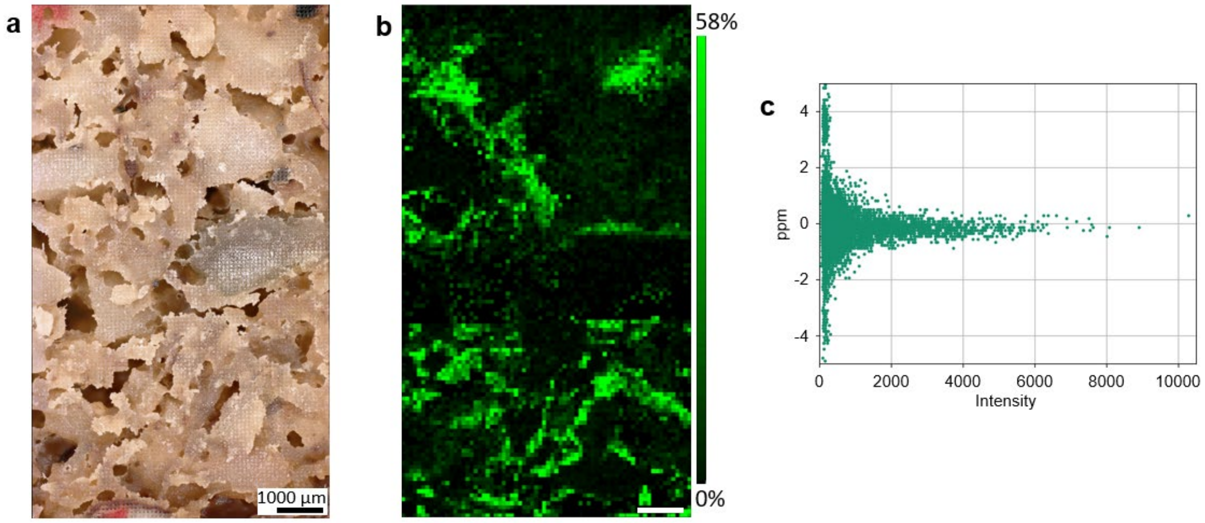

Fig. S6: MS imaging experiment of a gingerbread section cut with a knife. (**a**) optical image. (**b**) MS image of acrylamide $[M+H]^+$, *m/z* 72.04439, TIC-normalized. Image dimensions are 72x127 pixels with a step size of 90 µm and an *m/z* range of 70-280. (**c**) mass measurement accuracy plot of *m/z* 72.04439, RMSE = 0.67 ppm.

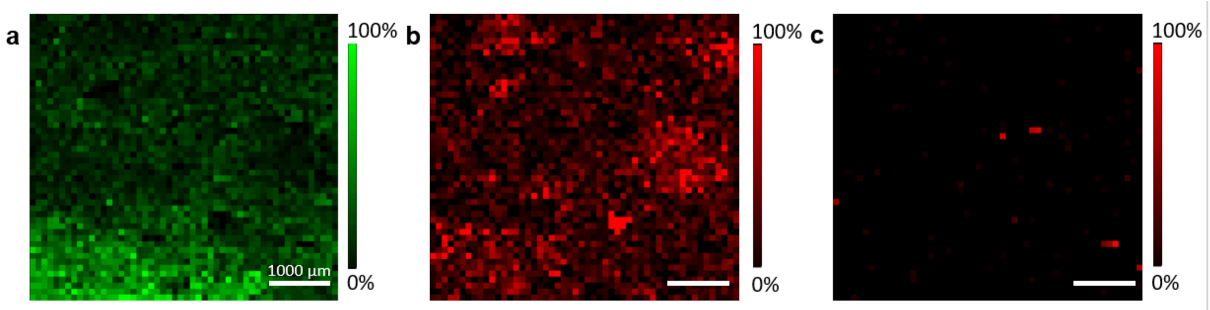

Fig. S7: In-situ derivatization of acrylamide on German gingerbread (measurement see Fig. S7). (**a**) MS image of acrylamide analogous to Fig. S7b. (**b**) Indexed MS image of TSA $[M+H]^+$, *m/z* 155.01613; $[M+Na]^+$, *m/z* 176.99807; $[M+K]^+$, *m/z* 192.97201 and $[M+NH_4]^+$, *m/z* 172.04268, TIC-normalized. (**c**) Indexed MS image of the Michael-adduct $[M+H]^+$, *m/z* 226.05324; $[M+Na]^+$, *m/z* 248.03519; $[M+K]^+$, *m/z* 264.00912 and $[M+NH_4]^+$, *m/z* 243.07979, TIC-normalized.

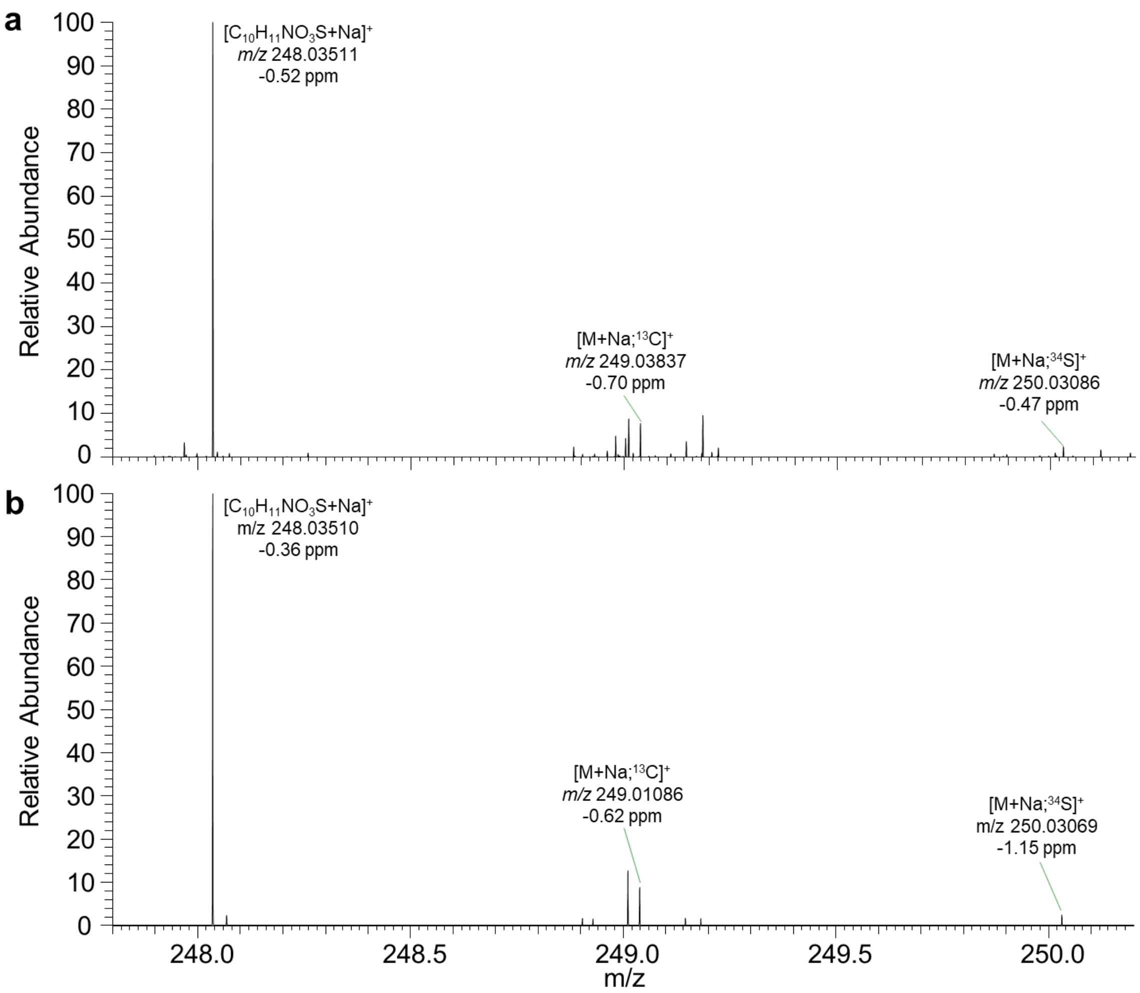

Fig. S8: Isotope distribution of the Michael-adduct, sum formula $[C_{10}H_{11}NO_2S+Na]^+$ (theoretical *m/z* 248.03519). (**a**) Average spectrum (n=102 single spectra) of the derivatization product formed from acrylamide standard on glass with sprayed TSA. (**b**) Single spectrum of the derivatization product formed on pancake sprayed with TSA and DHB.

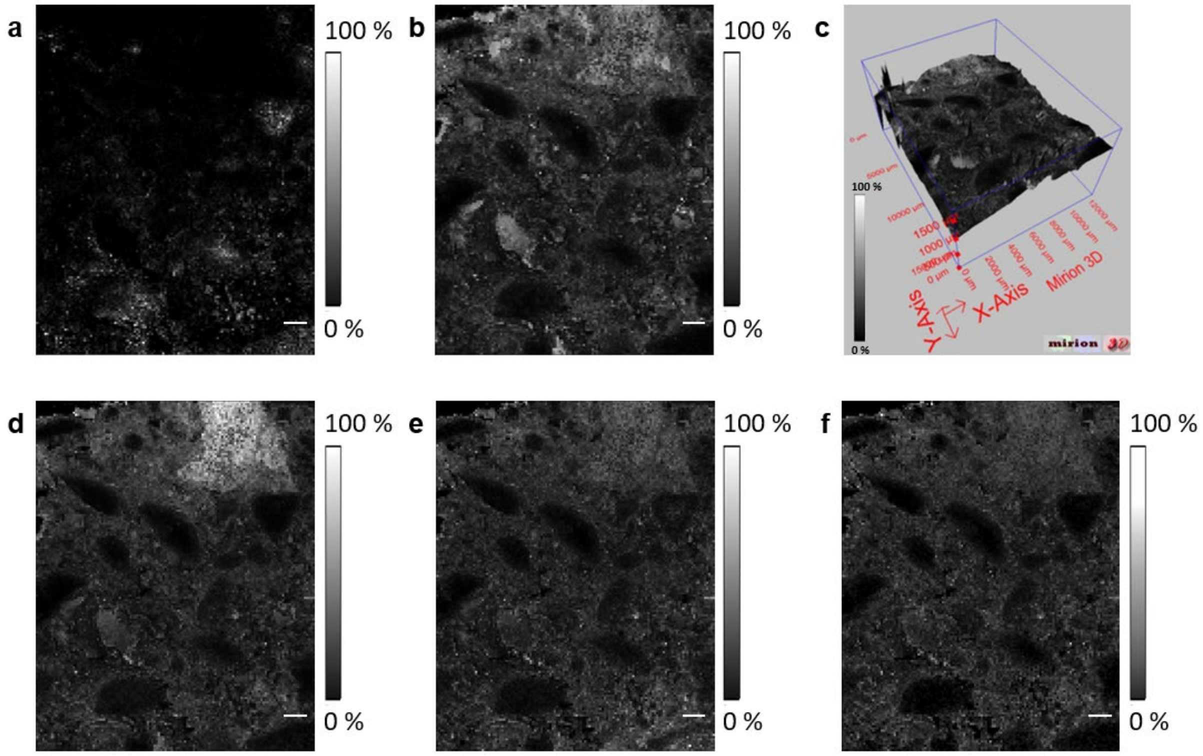

Fig. S9: Gray-scale MS images corresponding to Fig. 2. (**a**) MS image of PC 36:3 $[M+K]^+$, *m/z* 822.54096, TIC-normalized. (**b**) MS image of disaccharide $[M+K]^+$, *m/z* 381.07937, TIC-normalized. (**c**) 3D MS image of disaccharide $[M+K]^+$, *m/z* 381.07937, TIC-normalized, Z-axis is based on AF-laser focus data. (**d**) MS image of trisaccharide $[M+K]^+$, *m/z* 543.13219, TIC-normalized. (**e**) MS image of tetrasaccharide $[M+K]^+$, *m/z* 705.18502, TIC-normalized. (**f**) MS image of pentasaccharide $[M+K]^+$, *m/z* 867.23784, TIC-normalized.

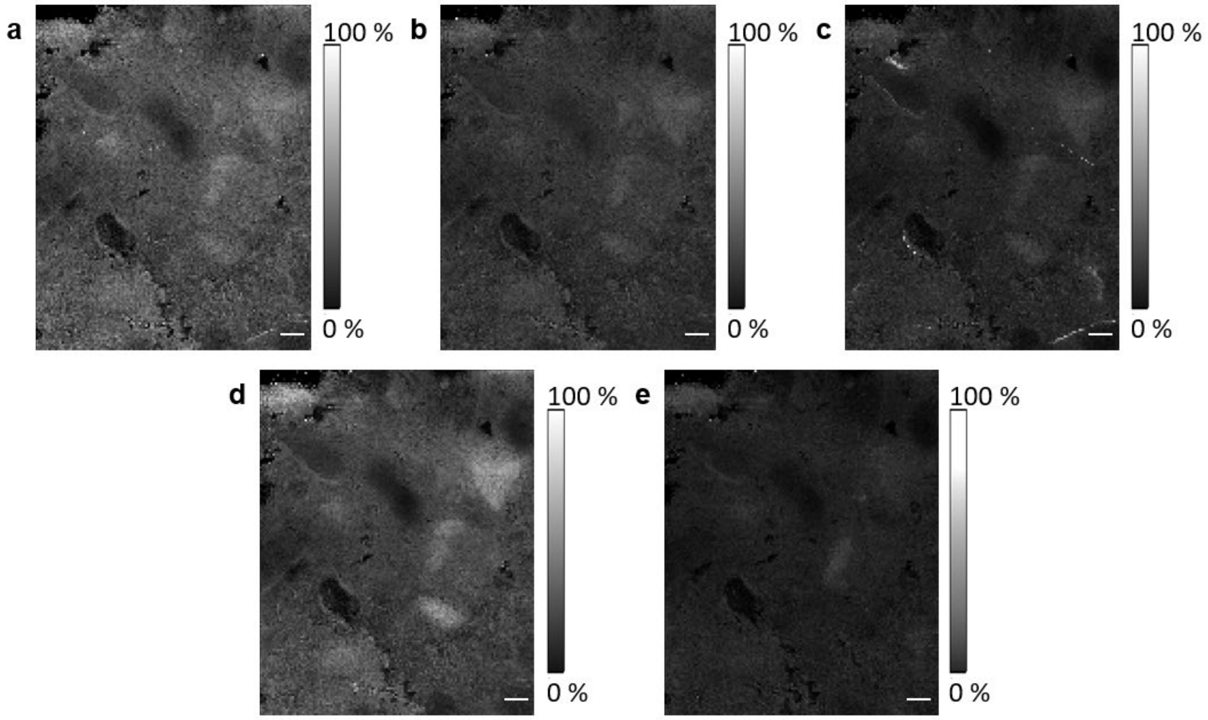

Fig. S10: Single-channel gray-scale MS images corresponding to Fig. S3. (**a**) MS image of TG 52:4 $[M+K]^+$, *m/z* 893.69950, TIC-normalized. (**b**) MS image of TG 52:3 $[M+K]^+$, *m/z* 895.71510, TIC-normalized. (**c**) MS image of TG 54:6 $[M+K]^+$, *m/z* 917.69950, TIC-normalized. (**d**) MS image of TG 54:5 $[M+K]^+$, *m/z* 919.71510, TIC-normalized. (**e**) MS image of TG 57:10 $[M+K]^+$, *m/z* 935.70990, TIC-normalized.
